# Author Correction: TDP-43 and PINK1 mediate *CHCHD10*^*S59L*^ mutation–induced defects in *Drosophila* and in vitro

**DOI:** 10.1038/s41467-021-23648-1

**Published:** 2021-05-20

**Authors:** Minwoo Baek, Yun-Jeong Choe, Sylvie Bannwarth, JiHye Kim, Swati Maitra, Gerald W. Dorn, J. Paul Taylor, Veronique Paquis-Flucklinger, Nam Chul Kim

**Affiliations:** 1 Department of Pharmacy Practice and Pharmaceutical Sciences, College of Pharmacy, University of Minnesota, Duluth, MN USA; 2 Inserm U1081, CNRS UMR7284, IRCAN, Université Côte d’Azur, CHU de Nice, Nice, France; 3 Center for Pharmacogenomics, Washington University School of Medicine, St. Louis, MO USA; 4 Howard Hughes Medical Institute and Department of Cell and Molecular Biology, St. Jude Children’s Research Hospital, Memphis, TN USA

**Keywords:** Mechanisms of disease, Mitochondria, Molecular neuroscience

Correction to: *Nature Communications* 10.1038/s41467-021-22145-9, published online 26 March 2021.

The original version of the Supplementary Information associated with this Article inadvertently omitted Supplementary Figure 9 due to an error during the revision process. The HTML has been updated to include a corrected version of the Supplementary Information.

## Supplementary information

Supplementary Information

