# Supplementary material for: TDP-43 and PINK1 mediate CHCHD10S59L mutation–induced defects in Drosophila and in vitro
Source: Nat Commun. 2021 Mar 26;12:1924. doi: 10.1038/s41467-021-22145-9 (PMC7997989; doi:10.1038/s41467-021-22145-9)
Supplement: Supplementary file 3 — Reporting Summary [file 41467_2021_22145_MOESM3_ESM.pdf]

nature research

Corresponding author(s): Nam Chul Kim

Last updated by author(s): 2/22/2021

Reporting Summary

Nature Research wishes to improve the reproducibility of the work that we publish. This form provides structure for consistency and transparency in reporting. For further information on Nature Research policies, see Authors & Referees and the Editorial Policy Checklist .

Statistics

For all statistical analyses, confirm that the following items are present in the figure legend, table legend, main text, or Methods section.

n/a	Confirmed	
☐	☒	The exact sample size (n) for each experimental group/condition, given as a discrete number and unit of measurement
☐	☒	A statement on whether measurements were taken from distinct samples or whether the same sample was measured repeatedly
☐	☒	The statistical test(s) used AND whether they are one- or two-sided<br>*Only common tests should be described solely by name; describe more complex techniques in the Methods section.*
☒	☐	A description of all covariates tested
☒	☐	A description of any assumptions or corrections, such as tests of normality and adjustment for multiple comparisons
☐	☒	A full description of the statistical parameters including central tendency (e.g. means) or other basic estimates (e.g. regression coefficient) AND variation (e.g. standard deviation) or associated estimates of uncertainty (e.g. confidence intervals)
☐	☒	For null hypothesis testing, the test statistic (e.g. F, t, r) with confidence intervals, effect sizes, degrees of freedom and P value noted<br>*Give P values as exact values whenever suitable.*
☒	☐	For Bayesian analysis, information on the choice of priors and Markov chain Monte Carlo settings
☒	☐	For hierarchical and complex designs, identification of the appropriate level for tests and full reporting of outcomes
☒	☐	Estimates of effect sizes (e.g. Cohen's d, Pearson's r), indicating how they were calculated

Our web collection on statistics for biologists contains articles on many of the points above.

Software and code

Policy information about availability of computer code

Data collection	Mina, https://github.com/ScienceToolkit/sciencetoolkit-mina.<br>Tracker (ver. 5.0.6, open source physics).<br>Ilastik (1.3.3post3).<br>Flynotyper 1.0<br>Huygens Essential software 20.04 (Scientific volume imaging)
Data analysis	ZEN 2.3 SP1 (Carl Zeiss).<br>Image Studio Lite Ver. 5.2.<br>Prism5 (Graphpad).<br>R (3.8.0, GNU General Public License)

For manuscripts utilizing custom algorithms or software that are central to the research but not yet described in published literature, software must be made available to editors/reviewers. We strongly encourage code deposition in a community repository (e.g. GitHub). See the Nature Research guidelines for submitting code & software for further information.

Data

Policy information about availability of data

All manuscripts must include a data availability statement. This statement should provide the following information, where applicable:
- Accession codes, unique identifiers, or web links for publicly available datasets
- A list of figures that have associated raw data
- A description of any restrictions on data availability

All Data generated in this study are available upon request from NCK. Source data are provided with this paper.

Field-specific reporting

Please select the one below that is the best fit for your research. If you are not sure, read the appropriate sections before making your selection.

- [x] Life sciences
- [ ] Behavioural & social sciences
- [ ] Ecological, evolutionary & environmental sciences

For a reference copy of the document with all sections, see nature.com/documents/nr-reporting-summary-flat.pdf

Life sciences study design

All studies must disclose on these points even when the disclosure is negative.

Sample size	The sample size was determined empirically based on previous experience and literature. For most cell biological and biochemical assays, we chose three biological replicates with three technical replicates as the minimum sample size. For Drosophila behavioral analyses, three independent measurements were performed with multiple flies and larvae as described in the manuscript.
Data exclusions	We did not exclude any data.
Replication	In most cases, we performed experiments with three biological replicates. For some fly experiments, data were collected from a single experiment because the results were obvious in numerous isogenic progenies and consistent with the initial screening. Mitochondrial network analysis in patient fibroblasts were repeated twice due to the apparent result from two independent patient fibroblasts.
Randomization	We did not randomize animals for behavioral assays because all assays were a group analysis with multiple isogenic animals. Other cell biological experiments do not require randomization.
Blinding	We did not use any blinding method in Drosophila experiments because expected results were obvious after initial screening. We also employed software-based quantitative analysis methods for all Drosophila experiments to avoid any possible human bias.

Reporting for specific materials, systems and methods

We require information from authors about some types of materials, experimental systems and methods used in many studies. Here, indicate whether each material, system or method listed is relevant to your study. If you are not sure if a list item applies to your research, read the appropriate section before selecting a response.

Materials & experimental systems

n/a	Involved in the study	
[ ]	[x]	Antibodies
[ ]	[x]	Eukaryotic cell lines
[x]	[ ]	Palaeontology
[ ]	[x]	Animals and other organisms
[x]	[ ]	Human research participants
[x]	[ ]	Clinical data

Methods

n/a	Involved in the study	
[x]	[ ]	ChIP-seq
[x]	[ ]	Flow cytometry
[x]	[ ]	MRI-based neuroimaging

Antibodies

Antibodies used

*Immunostaining:
FLAG (Sigma; #F1804 and Proteintech; #20543-1-AP, 1: 200),
FLAG-tagged Alexa Fluor 488 (Invitrogen; #MA1-142-A488, 1: 250),
HA (Cell Signaling; #3724 and Proteintech; #51064-2-AP, 1: 200),
Myc (Proteintech; #16286-2-AP, 1: 200),
V5 (Life Technology; #R96025, 1: 250),
CHCHD10 (Proteintech; 25617-1-AP, 1: 200),
CHCHD2 (Proteintech; 19424-1-AP, 1: 200)
TOM20 (Cell Signaling; #42460 and scbt; #sc-17764, 1: 250),
TDP43 (Proteintech; #60019-2-Ig and scbt; #sc-100871, 1: 200),
p-Ub (EMD milipore; #ABS15131, 1: 100),
Cy3-goat anti-Horseradish Peroxidase (Jackson ImmunoResearch Laboratories; #123-165-021, 1: 200)
4F3 anti-discs Large (Developmental Studies Hybridoma Bank; #4F3 anti-discs large; 1: 200)
Strepavidin, Alexa Flour 488 (Invitrogen; #S32354, 1: 250)
Alexa Fluor 594 phalloidin (Invitrogen; #A12381, 1: 250)
Alexa Fluor 488 goat anti-mouse IgG (Invitrogen; #A11001, 1: 250)
Alexa Fluor 594 goat anti-mouse IgG (Invitrogen; #A11005, 1: 250)
Alexa Fluor 633 goat anti-mouse IgG (Invitrogen; #A21050, 1: 250)

Alexa Fluor 488 goat anti-rabbit IgG (Invitrogen; #A11008, 1: 250)
Alexa Fluor 594 goat anti-rabbit IgG (Invitrogen; #A11012, 1: 250)
Alexa Fluor 633 goat anti-rabbit IgG (Invitrogen; #A21070, 1: 250)

*Western blot:
FLAG (Sigma; #F1804 and Proteintech; #20543-1-AP, 1: 1,000),
HA (Cell Signaling; #3724 and Proteintech; #51064-2-AP, 1: 1,000),
Myc (Proteintech; #16286-2-AP, 1: 1,000),
HSP60 (Cell signaling; #4870, 1: 1,000),
CHCHD10 (Proteintech; 25617-1-AP, 1: 1,000),
CHCHD2 (Proteintech; 19424-1-AP, 1: 1,000)
Cox2 (Abcam; #ab-79393, 1: 2,000),
LC3B (Cell signaling; #2775, 1: 1,000),
LAMP1 (Cell signaling; #9091, 1: 1,000),
p-Ub (EMD milipore; #ABS15131,1: 1000),
Actin (SCBT; #sc-47778 and Proteintech; #20536-1-AP, 1: 3,000),
PINK1 (SCBT; #sc-517353, 1: 500, Novus; #BC100-494, 1: 1,000),
Drp1 (Cell signaling; #8570, 1: 1,000),
Mfn1 (Cell signaling; #14739, 1: 1,000),
Mfn2 (Cell signaling; #11925, 1: 1,000),
NDP52 (Proteintech; #12229-1-AP, 1: 1,000),
OPTN (Proteintech; #10837-1-AP, 1: 1,000),
IRDye 680RD Goat anti-mouse (Li-cor; #926-68070, 1: 5,000)
IRDye 800CW Donkey anti-mouse (Li-cor; #926-32212, 1: 5,000)
IRDye 680RD Goat anti-rabbit (Li-cor; #926-68071, 1: 5,000)
IRDye 800CW Donkey anti-rabbit (Li-cor; #926-32213, 1: 5,000)

*Immunoprecipitation:
EZview anti-FLAG M2 affinity Gel (Sigma; #F2426),
EZview anti-HA affinity gel (Sigma; #E6779)

Validation

All antibodies have been validated by the manufacturer and produced expected results in our experiments.
Optimal antibody concentrations used in our experiments are indicated in this report summery and the method section of the manuscript.

Eukaryotic cell lines

Policy information about cell lines

Cell line source(s)

HeLa (ATCC, CCL-2), SH-SY5Y (ATCC, CRL-2266) and HEK293T (ATCC, CRL-3216) purchased from American Type Culture Collection (ATCC). YFP-Parkin stably expressing HeLa and matched control HeLa (Dr. Richard Youle, NIH), Pink1-V5-His stable HeLa and matched control HeLa cell (Dr. Richard Youle, NIH). Pink1 KO HeLa and matched control HeLa (Dr. Wade Harper, Harvard).

Authentication

None of the cell lines has not been authenticated again after getting from ATCC and individual researchers one or two years ago.

Mycoplasma contamination

Mycoplasma contamination has not yet been performed after getting cell lines.

Commonly misidentified lines
(See ICLAC register)

No commonly misidentified cell lines are used in the study.

Animals and other organisms

Policy information about studies involving animals; ARRIVE guidelines recommended for reporting animal research

Laboratory animals

Drosophila melanogaster

Wild animals

This study did not involve wild animals

Field-collected samples

This study did not involve field collected samples.

Ethics oversight

No ethical approval is required for Drosophila studies.

Note that full information on the approval of the study protocol must also be provided in the manuscript.
