# Supplementary material for: TDP-43 and PINK1 mediate CHCHD10S59L mutation–induced defects in Drosophila and in vitro
Source: Nat Commun. 2021 Mar 26;12:1924. doi: 10.1038/s41467-021-22145-9 (PMC7997989; doi:10.1038/s41467-021-22145-9)
Supplement: Supplementary file 1 — Supplementary Information [file 41467_2021_22145_MOESM1_ESM.pdf]

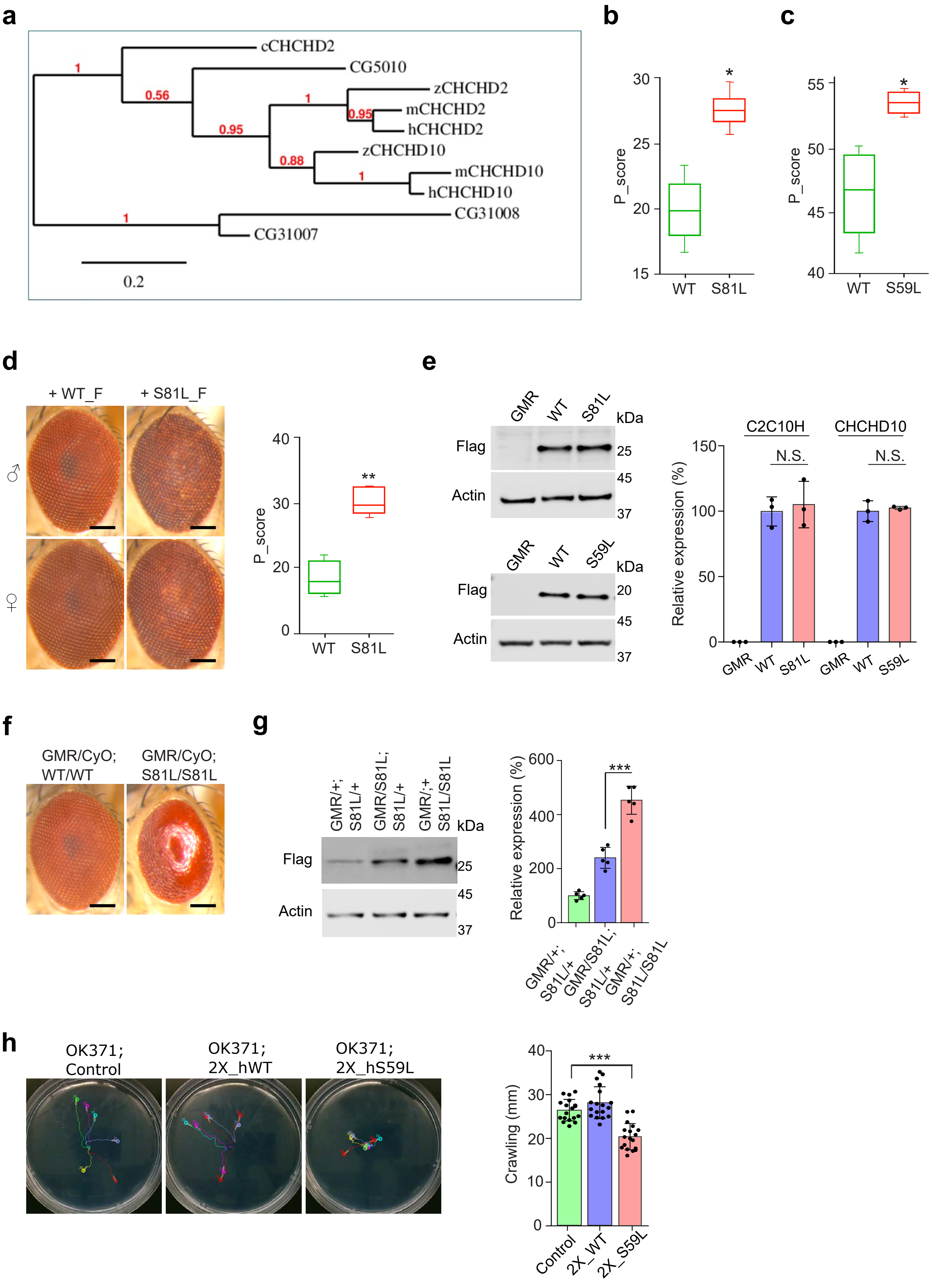

a
cCHCHD2
CG5010
zCHCHD2
mCHCHD2
hCHCHD2
zCHCHD10
mCHCHD10
hCHCHD10
CG31008
CG31007
1
0.56
0.95
1
0.95
0.88
1
1
0.2
b
P_score
WT
S81L
*
c
P_score
WT
S59L
*
d
+ WT_F
+ S81L_F
♂
♀
P_score
**
WT
S81L
e
GMR
WT
S81L
kDa
Flag
Actin
GMR
WT
S59L
Flag
Actin
C2C10H
CHCHD10
N.S.
N.S.
Relative expression (%)
GMR
WT
S81L
GMR
WT
S59L
f
GMR/CyO; WT/WT
GMR/CyO; S81L/S81L
g
GMR/+; S81L/+
GMR/S81L; S81L/+
GMR/;+ S81L/S81L
kDa
Flag
Actin
Relative expression (%)
***
GMR/+; S81L/+
GMR/S81L; S81L/+
GMR/+; S81L/S81L
h
OK371; Control
OK371; 2X_hWT
OK371; 2X_hS59L
Crawling (mm)
***
Control
2X_WT
2X_S59L

Supplementary Fig. 1. Phenotypes of *Drosophila* expressing *C2C10H* and *CHCHD10.* (**a**) Phylogenetic tree for *CHCHD2* and *CHCHD10*. The genetic tree was generated with Phylogeny.fr. (**b and c**) Degenerative eye quantification shown in Fig. 1b and Fig. 1c, respectively. Boxes indicate medians and 25th and 75th percentiles. Bars indicate the highest and lowest values (two-sided *t*-test,**p* = 0.0448 (Fig. 1b) and 0.0291 (Fig. 1c); *n* = 4 flies from 2 independent expeirments (Fig. 1b) and n=5 flies from 2 independent experiments (Fig. 1c)). Note that p-scores cannot be directly compared between Fig. 1b and Fig. 1c (see Methods). The p-scores of normal GMR-GAL4 for Fig. 1b and Fig. 1c were 23.12 ± 1.5 (*n* = 5) and 46.37 ± 3.8 (*n* = 7), respectively. (**d**) FLAG-tagged $C2C10H^{S81L}$ causes age-dependent rough eye phenotypes in 40-day-old flies. Boxes indicate medians and 25th and 75th percentiles. Bars indicate the highest and lowest values (two-sided *t*-test, ***p* = 0.0023; *n* = 5 for each group). Scale bar = 200 μm. (**e**) Proteins extracted from heads expressing FLAG-tagged *C2C10H WT*, *S81L* (upper) and FLAG-tagged *CHCHD10 WT* and *S59L* (lower) by *GMR-GAL4* and immunoblotting with anti-FLAG and anti-actin antibodies was performed. Data are mean ± SD (two-sided *t*-test, N.S., not significant; *n* = 3 independent experiments). (**f**) Expression from third chromosome homozygous $C2C10H^{S81L}$ at the attp2 site (homozygote) causes a severe rough eye phenotype at eclosion. Scale bar = 200 μm. (**g**) To confirm the increased expression of $\mathrm{C2C10H^{S81L}}$ protein via transvection, proteins were extracted from heads, and immunoblotting was conducted with anti-FLAG and anti-actin antibodies. As shown and quantified, fly eyes carrying two homozygous copies of $C2C10H^{S81L}$ at the attp2 site (GMR/+;S81L/S81L) expressed a significantly higher amount of C2C10H than fly eyes carrying two heterozygous copies of $C2C10H^{S81L}$ at the attp40 and attp2 site (GMR/S81L;S81L/+). However, fly eyes carrying two heterozygous copies of $C2C10H^{S81L}$ (GMR/S81L;S81L/+) expressed approximately twice the amount of C2C10H than

fly eyes carrying one copy of *C2C10H*^{S81L} (GMR/+;S81L/+) as expected. Data are mean ± SD (one-way ANOVA and *posthoc* Dunnett test, two-sided, ***p = 8.005e-05; n = 5). (**h**) Expression of *CHCHD10*^{S59L} in motor neurons results in defective locomotive activity. The locomotive activity was assessed by the crawling distance of third-instar larvae. Data are mean ± SD (one-way ANOVA and *posthoc* Dunnett test, two-sided, ***p = 8.2e-06; n = 18).

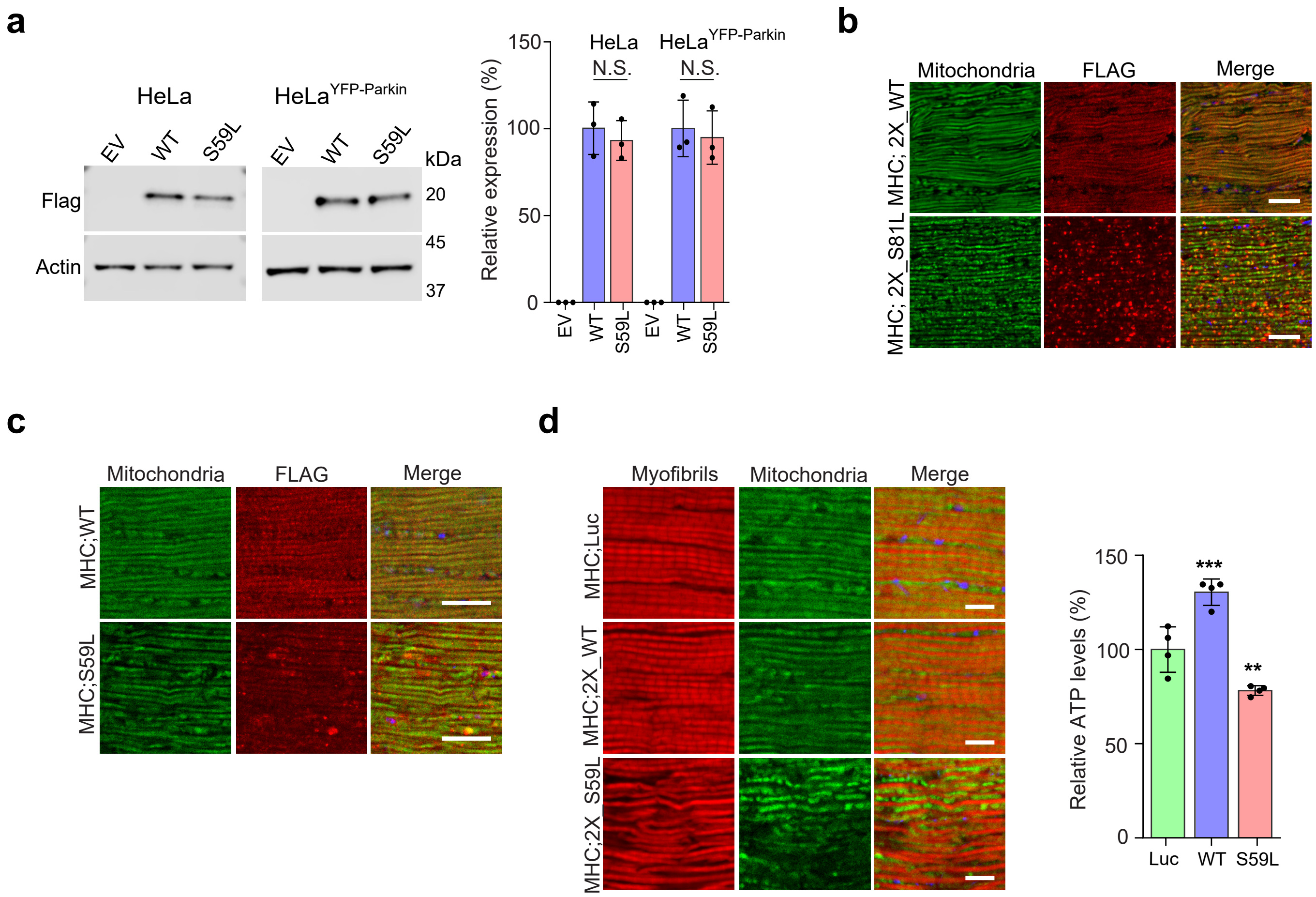
a
HeLa
HeLa YFP-Parkin
EV
WT
S59L
kDa
Flag
Actin
20
45
37
Relative expression (%)
150
100
50
0
N.S.
b
Mitochondria
FLAG
Merge
MHC; 2X_WT
MHC; 2X_S81L
c
MHC;WT
MHC;S59L
d
Myofibrils
MHC;Luc
MHC;2X_WT
MHC;2X_S59L
Relative ATP levels (%)
***
**
Luc

Supplementary Fig. 2 Expression of *CHCHD10^{WT}* and *CHCHD10^{S59L}* constructs in HeLa and HeLa$^{YFP\text{-}Parkin}$ cells, the aggregate formation of C2C10H^{S81L} and CHCHD10^{S59L}, and mitochondrial defects caused by CHCHD10^{S59L} in *Drosophila* muscle. (**a**) Expression of FLAG-tagged *CHCHD10^{WT}* and *CHCHD10^{S59L}* in HeLa and HeLa$^{YFP\text{-}Parkin}$ cells. HeLa cells were transiently transfected and subjected to immunoblotting with anti-FLAG and anti-actin antibodies. Data are mean ± SD (two-sided *t*-test, N.S., not significant; n = 3 independent experiments). (**b**) Indirect flight muscles from 10-day-old *MHC*-GAL4>*UAS*-2X_*C2C10H^{WT}* and 2X_*C2C10H^{S81L}* male flies were immunostained with streptavidin–Alexa Fluor 488 (green) and anti-FLAG antibody (red) to visualize mitochondria and C2C10H^{WT} or C2C10H^{S81L}, respectively. Representative images from 2 independent experiments with 9 flies are shown. Scale bar = 20 μm. (**c**) Indirect flight muscles from 20-day-old *MHC*-GAL4>*UAS-CHCHD10^{WT}* and *CHCHD10^{S59L}* males were immunostained with streptavidin–Alexa Fluor 488 (green) and anti-FLAG antibody (red) to visualize mitochondria and CHCHD10^{WT} or CHCHD10^{S59L}, respectively. Scale bar = 20 μm. (**d**) Muscular and mitochondrial morphology of 20-day-old *CHCHD10^{WT}*- and *CHCHD10^{S81L}*-expressing flies. Indirect flight muscles were stained with streptavidin–Alexa Fluor 488 (mitochondria, green) and phalloidin–Alexa Fluor 594 (actin filaments, red). Representative images from 2 independent experiments with 10 flies are shown. ATP levels in thoraxes containing muscle tissues of the indicated genotypes (aged 20 days) were measured. Data shown are mean ± SD (one-way ANOVA and *posthoc* Dunnett test, two-sided, ***p = 0.00097 and **p = 0.00821; n = 4 independent experiments). Scale bar = 10 μm.

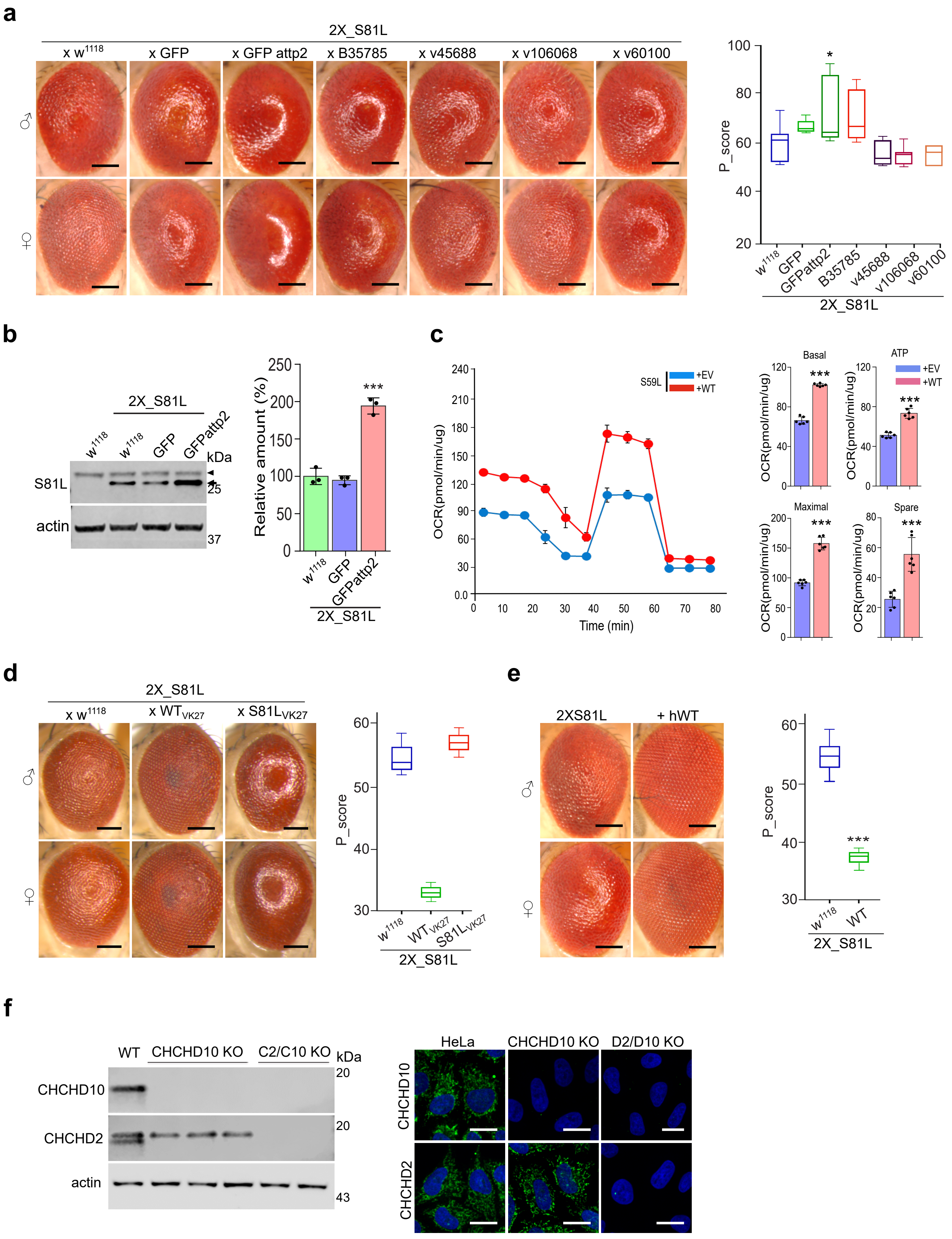
a
2X_S81L
x w1118
x GFP
x GFP attp2
x B35785
x v45688
x v106068
x v60100
♂
♀
P_score
100
80
60
40
20
*
w1118
GFP
GFPattp2
B35785
v45688
v106068
v60100
2X_S81L
b
2X_S81L
w1118
w1118
GFP
GFPattp2
kDa
S81L
25
actin
37
Relative amount (%)
250
200
150
100
50
0
***
w1118
GFP
GFPattp2
2X_S81L
c
OCR(pmol/min/ug)
240
210
180
150
120
90
60
30
0.0
S59L
+EV
+WT
0
10
20
30
40
50
60
70
80
Time (min)
Basal
ATP
Maximal
Spare
***
d
2X_S81L
x w1118
x WTVK27
x S81LVK27
♂
♀
P_score
60
50
40
30
w1118
WTVK27
S81LVK27
2X_S81L
e
2XS81L
+ hWT
♂
♀
P_score
60
50
40
30
***
w1118
WT
2X_S81L
f
WT
CHCHD10 KO
C2/C10 KO
kDa
CHCHD10
20
CHCHD2
20
actin
43
HeLa
CHCHD10 KO
D2/D10 KO
CHCHD10
CHCHD2

g

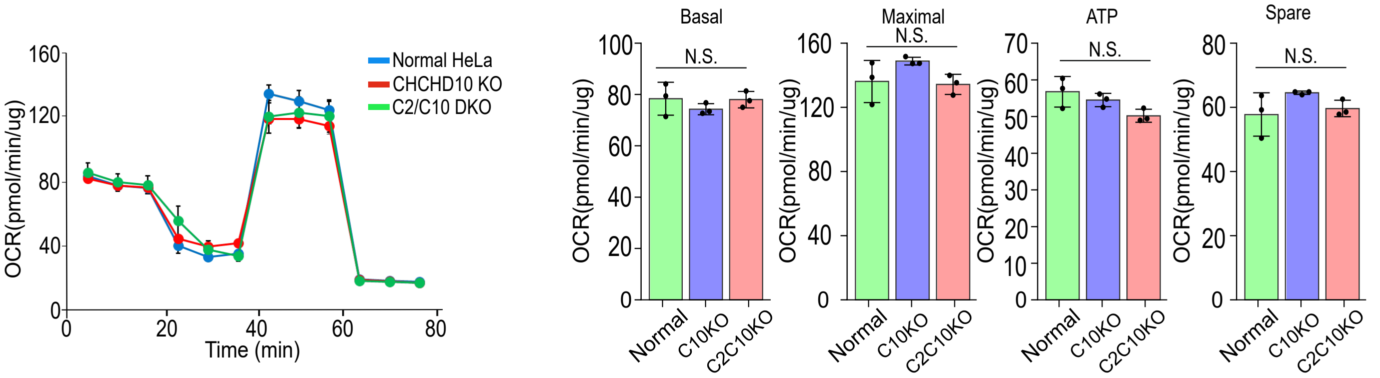

Normal HeLa
CHCHD10 KO
C2/C10 DKO
OCR(pmol/min/ug)
Time (min)
Basal
Maximal
ATP
Spare
N.S.
Normal
C10KO
C2C10KO

h

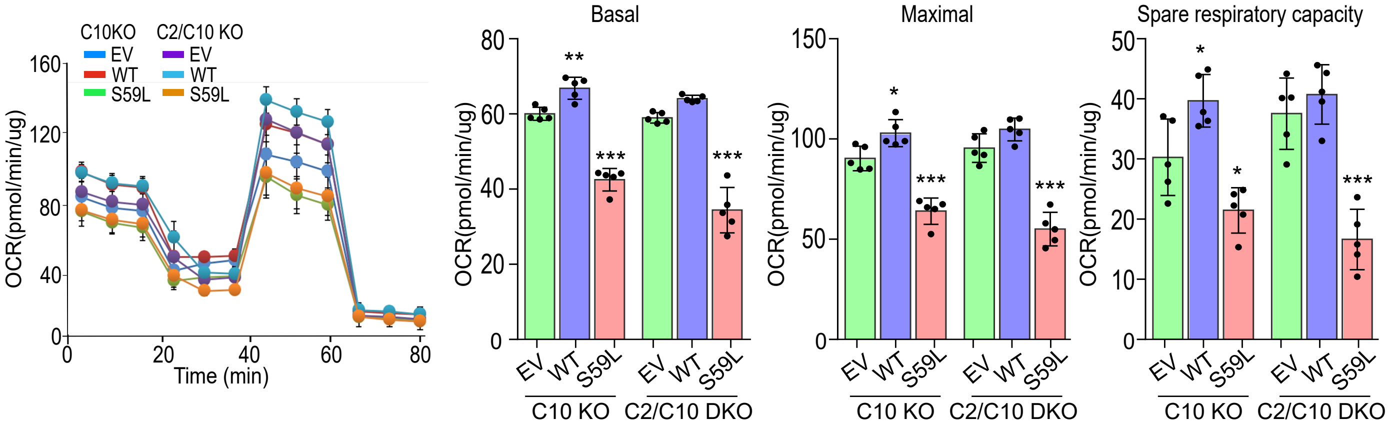

C10KO
C2/C10 KO
EV
WT
S59L
OCR(pmol/min/ug)
Time (min)
Basal
Maximal
Spare respiratory capacity
**
***
*
C10 KO
C2/C10 DKO

Supplementary Fig. 3 Co-expression of *CHCHD10^{WT}* and *CHCHD10^{S59L}* in *Drosophila* and HeLa cells. (**a**) Eye phenotypes of various control lines in the 2X_*C2C10H^{S81L}* background. GFP (B1512), p element–mediated insertion on second chromosome; GFPattp2, GFP inserted on attp2 site; B35785, TRiP mCherry RNAi control; V45688, VDRC GD RNAi line (ebony); V106080, VDRC KK RNAi line (yellow); and V60100, host line of KK lines. Representative images from 3 independent experiments are shown. Boxes indicate median and 25th and 75th percentiles, and bars indicate the highest and lowest values (one-way ANOVA and *posthoc* Dunnett test, two-sided, *p = 0.02952; n = 5–8 for each group). Control lines carrying a transgene at the attp2 site showed slightly enhanced rough eyes due to transvection, but other control lines did not induce notable modification. Because GFP (B1512) showed a trend, although not statistically significant, for enhanced rough eye phenotypes, we measured CHCHD10 protein levels. As shown in (b and b′), only GFPattp2 inserted at the attp2 site increased CHCHD10 expression significantly. Scale bar = 200 μm. (**b**) Proteins were extracted from the heads of w^{1118}, GFP, and GFPattp2 flies in the 2X_C2C10H^{S81L} background, and then immunoblotting was performed with anti-FLAG and anti-actin antibodies (arrow and arrowhead indicate S81L and non-specific bands, respectively). Data are mean ± SD (one-way ANOVA and *posthoc* Dunnett test, two-sided, ***p = 3.4e-05; n = 3 independent experiments). (**c**) HeLa cells were co-transfected with FLAG-tagged *CHCHD10^{S59L}* and *CHCHD10^{WT}*. Empty vector (EV) was used as a control. After 24 hours, mitochondrial respiration for each group was measured by Seahorse XF Cell Mito Stress tests. Graphs of a single representative experiment are shown (mean ± SD). Actual statistical analyses were performed with 8 independent experiments (one-way ANOVA and *posthoc* Dunnett test, two-sided, ***p = 8.01e-10, 7.52e-08, 2.38e-06 and 0.000156 for basal, maximal, ATP and spare level, respectively; detailed information on statistical analyses is

available in Supplementary Fig. 9d). (**d**) Expression of *C2C10H*^{WT} inserted in the *VK27* site also improved *C2C10H*^{S81L}-induced rough eye phenotypes, whereas insertion of *C2C10H*^{S81L} in the *VK27* site exacerbated the phenotypes. Boxes indicate median and 25th and 75th percentiles, and bars indicate the highest and lowest values (n = 2 from a single experiment). Scale bar = 200 µm. (**e**). Expression of human *CHCHD10*^{WT} mitigated *C2C10H*^{S81L}-induced rough eye phenotypes. Boxes indicate median and 25th and 75th percentiles, and bars indicate the highest and lowest values (two-sided t-test, ***p = 2.04e-05, n = 7-10 flies from 2 independent experiment). Scale bar = 200 µm. (**f**) Lysates from HeLa cells, three *CHCHD10*^{KO} HeLa cell lines, two *CHCHD2/CHCHD10* double knockout HeLa cell lines (C2/C10 DKO) were immunoblotted with anti-CHCHD10 and anti-CHCHD2 antibodies and immunostained. Representative images from 3 independent experiments are shown. Scale bar = 20 µm. (**g**). Mitochondrial respiration of normal HeLa, *CHCHD10*^{KO} HeLa, and *CHCHD2/10*^{DKO} HeLa cell lines was measured by Seahorse XF Cell Mito Stress tests. Graphs of a single representative experiment were shown (mean ± SD). Actual statistical analyses were performed with 3 independent experiments (two-sided t-test; detailed information on statistical analyses is available in Supplementary Fig. 9e). (**h**) EV, FLAG tagged *CHCHD10*^{WT}, and *CHCHD10*^{S59L} were transfected to *CHCHD10*^{KO} HeLa and *CHCHD2/10*^{DKO} HeLa cell lines. Mitochondrial respiration of *CHCHD10*^{KO} HeLa and *CHCHD2/10*^{DKO} HeLa cell lines was measured by Seahorse XF Cell Mito Stress tests. Graphs of a single representative experiment are shown (mean ± SD). Actual statistical analyses were performed with 3 independent experiments (one-way ANOVA and *posthoc* Dunnett test, two-sided, Basal: **p = 0.0027, ***p = 3.9e-07 in C10 KO and ***p = 3.0e-07 in C2/C10 DKO; Maximal: *p = 0.0177, ***p = 6.4e-05 in C10 KO and ***p =2.3e-06 in C2/C10 DKO; Spare

respiratory capacity: *p = 0.0203 for WT, 0.0287 for S59L in C10 KO, ***p = 8.5e-05 in C2/C10KO; detailed information on statistical analyses is available in Supplementary Fig. 9b, c).

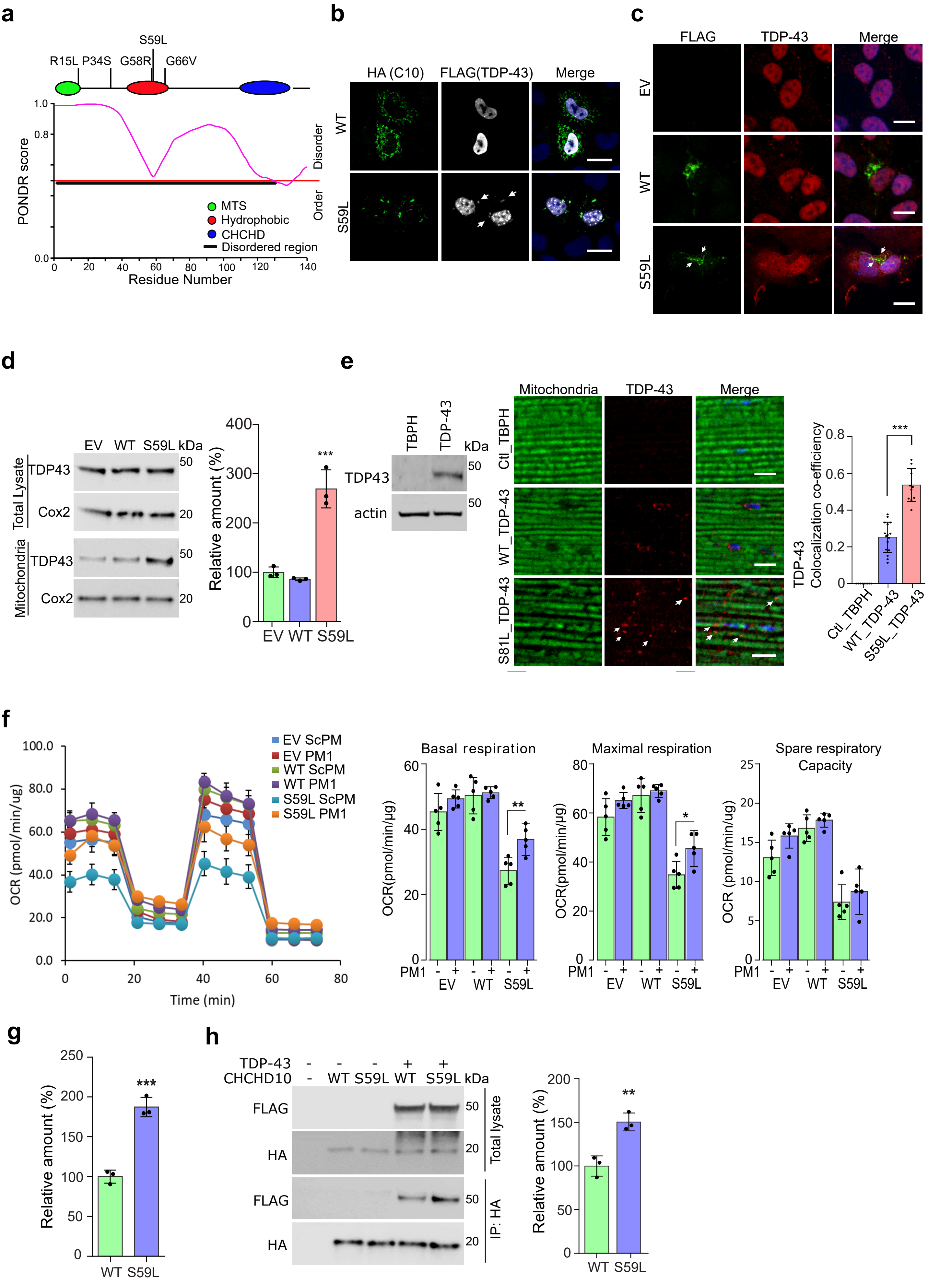
a
b
c
R15L P34S G58R S59L G66V
PONDR score
Residue Number
Disorder
Order
MTS
Hydrophobic
CHCHD
Disordered region
HA (C10)
FLAG(TDP-43)
Merge
WT
S59L
FLAG
TDP-43
EV
d
e
EV WT S59L kDa
Total Lysate
Mitochondria
TDP43
Cox2
Relative amount (%)
***
TBPH
TDP-43
actin
Mitochondria
Ctl_TBPH
WT_TDP-43
S81L_TDP-43
TDP-43 Colocalization co-efficiency
S59L_TDP-43
f
EV ScPM
EV PM1
WT ScPM
WT PM1
S59L ScPM
S59L PM1
OCR (pmol/min/ug)
Time (min)
Basal respiration
Maximal respiration
Spare respiratory Capacity
OCR(pmol/min/μg)
OCR (pmol/min/ug)
PM1
**
*
g
h
TDP-43
CHCHD10
FLAG
HA
Total lysate
IP: HA

Supplementary Fig. 4 Mitochondrial localization and functional effects of TDP-43 in *Drosophila* and human cells. (**a**) The intrinsically disordered region of CHCHD10 was predicted by PONDR. (**b**) HeLa cells were co-transfected with FLAG-tagged *TARDBP* and HA-tagged $CHCHD10^{WT}$ or $CHCHD10^{S59L}$. After 24 hours, cells were stained with anti-FLAG and anti-HA antibodies. Arrows indicate mitochondrial localization of TDP-43. Representative images from 2 independent experiments are shown. Scale bar = 20 μm. (**c**) SH-SY5Y cells were transfected with FLAG-tagged $CHCHD10^{WT}$ or $CHCHD10^{S59L}$. Cells were stained with anti-FLAG and anti-TDP-43 antibodies. Note that endogenous TDP-43 is highly distributed in mitochondria and cytoplasm of $\mathrm{CHCHD10^{S59L}}$-transfected cells compared to those of empty vector (EV), WT-transfected cells. Arrows indicate TDP-43 co-localized with mitochondria. Representative images from 4 independent experiments are shown. Scale bar = 10 μm. (**d**) SH-SY5Y cells were transfected with FLAG-tagged $CHCHD10^{WT}$ or $CHCHD10^{S59L}$. After 48 hours, mitochondria were isolated. Immunoblotting was conducted with anti-TDP-43 and anti-Cox2 antibodies. Data are shown mean ± SD (one-way ANOVA and *posthoc* Dunnett test, two-sided, ****p* = 0.0002, compared to empty vector (EV) control; *n* = 3 independent experiments). (**e**) Normal flies and humanized TDP-43 humanized flies were analyzed by immunoblot with anti-TDP-43 antibodies to confirm the expression of human TDP-43 in the humanized TDP-43 flies (left panel). Indirect flight muscles from flies expressing $C2C10H^{WT}$ or $C2C10H^{S81L}$ with human TDP-43 replacing the entire coding region of *TBPH*. Tissues were immunostained with streptavidin–Alexa Fluor 488 (green) and anti-TDP-43 antibody (red). Co-localization was measured by Mander correlation coefficient. Data are shown mean ± SD (two-sided *t*-test, ****p* = 1.41e-07; *n* = 10). Scale bar = 20 μm. (**f**) Mitochondrial respiration was measured by Seahorse XF Cell Mito Stress tests. Graphs of a single representative experiment are shown (mean ± SD).

Actual statistical analyses were performed with 4 independent experiments (one-way ANOVA and *posthoc* Dunnett test, two-sided, *$p = 0.0162$, **$p = 0.0064$; detailed information on statistical analyses is available in Supplementary Fig. 9f). (**g**) Quantification of Fig. 4i (two-sided *t*-test, ***$p = 0.000523$; $n = 3$ independent experiments). (**h**) HEK293T cells were transfected with/without *TDP-43-FLAG* and *CHCHD10*^{WT}*-HA* or *CHCHD10*^{S59L}*-HA*. After 24 hours, lysates were subjected to co-immunoprecipitation with anti-HA affinity gel, and immunoblotting was performed with anti-HA (CHCHD10) and anti-FLAG (TDP-43) antibodies. Note that CHCHD10$^{\mathrm{S59L}}$ protein binds TDP-43 more greatly than CHCHD10^{WT} protein (two-sided *t*-test, **$p = 0.004873$; $n = 3$ independent experiments).

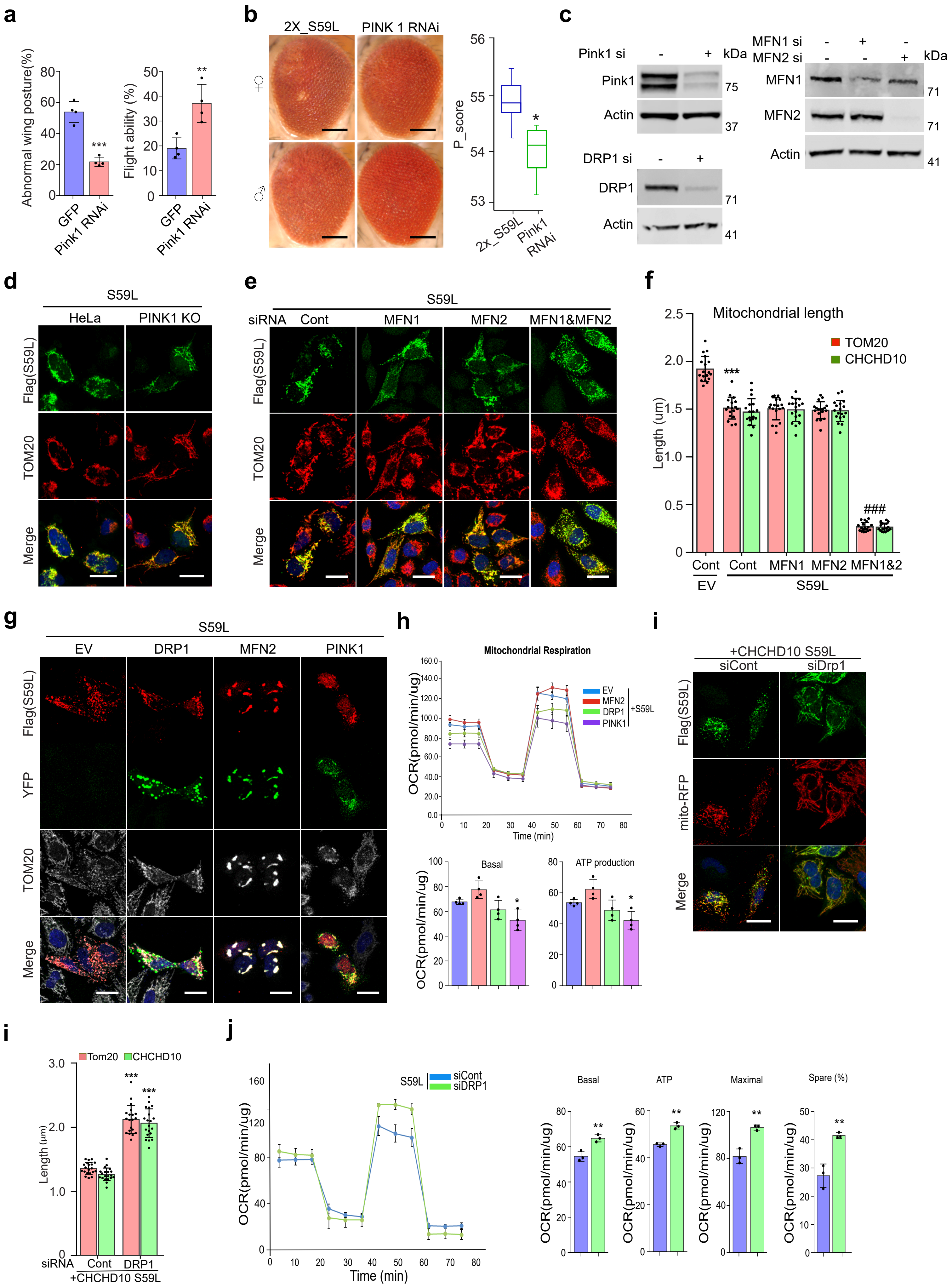

a
Abnormal wing posture(%)
Flight ability (%)
GFP
Pink1 RNAi
b
2X_S59L
PINK 1 RNAi
P_score
c
Pink1 si
Pink1
Actin
DRP1 si
DRP1
MFN1 si
MFN2 si
MFN1
MFN2
kDa
d
S59L
HeLa
PINK1 KO
Flag(S59L)
TOM20
Merge
e
siRNA
Cont
MFN1
MFN2
MFN1&MFN2
f
Mitochondrial length
TOM20
CHCHD10
Length (um)
EV
g
EV
DRP1
PINK1
YFP
h
Mitochondrial Respiration
OCR(pmol/min/ug)
Time (min)
+S59L
Basal
ATP production
i
+CHCHD10 S59L
siCont
siDrp1
mito-RFP
Tom20
+CHCHD10 S59L
j
siDRP1
ATP
Maximal
Spare (%)

Supplementary Fig. 5 Role of the PINK1/parkin pathway on the *CHCHD10*^{S59L}-induced phenotype. (**a**) Downregulation of *PINK1* mitigated abnormal wing postures and locomotor defects assessed by flight ability. Data are mean ± SD (two-sided *t*-test, ***p = 0.00013, **p = 0.0062; n = 4, with > 40 flies for each group). (**b**) RNAi-mediated knockdown effect of *PINK1* on human CHCHD10^{S59L}-induced eye phenotypes. Boxes indicate median and 25th and 75th percentiles, and bars indicate the highest and lowest values (two-sided *t*-test, *p = 0.0226; n = 8 flies from 3 independent experiments). Scale bar = 200 μm. (**c**) HeLa cells were transfected with siRNAs targeting *PINK1*, *DRP1*, *MFN1*, or *MFN2* or control siRNA. Immunoblotting confirmed the successful knockdown of target genes. Representative images from 3 independent experiments are shown. (**d**) HeLa cells and *PINK1*^{KO} HeLa cells were transfected with FLAG-tagged *CHCHD10*^{S59L} or empty vector (EV). Representative images (single experiments) of transfected HeLa cells were immunostained with antibodies against FLAG (green, CHCHD10) and TOM20 (red, mitochondria). Scale bar = 20 μm. (**e**) HeLa cells were transfected with siRNAs targeting *MFN1* and/or *MFN2*. The cells were transfected with FLAG-tagged *CHCHD10*^{S59L} 24 hours after siRNA transfection. Representative images (3 independent experiments) of transfected HeLa cells immunostained with antibodies against FLAG (green, CHCHD10) and TOM20 (red, mitochondria). Scale bar = 20 μm. (**f**) Quantification of TOM20 and CHCHD10^{S59L}-FLAG signal length. Data shown are mean ± SD (one-way ANOVA and Tukey-Kramer test, two-sided, ***p < 2e-16 *vs.* EV; n = 18-20 cells from 3 independent experiments; ###p < 2e-16 *vs.* *CHCHD10*^{S59L} control (Cont) cells). (**g**) HeLa cells were co-transfected with YFP-tagged *DRP1*, *MFN2*, or *PINK1* and FLAG-tagged *CHCHD10*^{S59L}. Transfected HeLa cells were immunostained with an antibody against FLAG (red, CHCHD10) and TOM20 (gray, mitochondria). Representative images (single experiment) are shown. Scale

bar = 20 µm. (**h**) Mitochondrial respiration was measured by Seahorse XF Cell Mito Stress tests 24 hours after transfection. Graphs of a single representative experiment are shown (mean ± SD). Actual statistical analyses were performed with 6 independent experiments (one-way ANOVA and *posthoc* Dunnett test, two-sided, *p = 0.0235 for basal level and 0.0293 for ATP production; detailed information on statistical analyses is available in Supplementary Fig. 9h). (**i**) HeLa cells were transfected with a siRNA targeting *DRP1*. After 18 hours of siRNA transfection, the cells were transfected with FLAG-tagged $CHCHD10^{S59L}$. Representative images (3 independent experiments) of transfected HeLa cells immunostained with antibodies against FLAG (green, CHCHD10) and TOM20 (red, mitochondria). Quantification of TOM20 and $\text{CHCHD10}^{\text{S59L}}$-FLAG signal length. Green $\text{CHCHD10}^{\text{S59L}}$ signals are spread across mitochondria. Data shown are mean ± SD (two-sided t-test, ***p = 8.32e-17 for Tom20 and 3.72e-17 for CHCHD10 *vs.* EV; n = 20 cells from 3 independent experiments). Scale bar = 20 µm. (**j**) Mitochondrial respiration was measured by Seahorse XF Cell Mito Stress tests 24 hours after transfection. Graphs of a single representative experiment are shown (mean ± SD). Actual statistical analyses were performed with 3 independent experiments (two-sided, t-test, **p = 0.00753, 0.00312, 0.00689 and 0.0045 for basal, maximal, ATP and spare (%), respectively; detailed information on statistical analyses is available in Supplementary Fig. 9i).

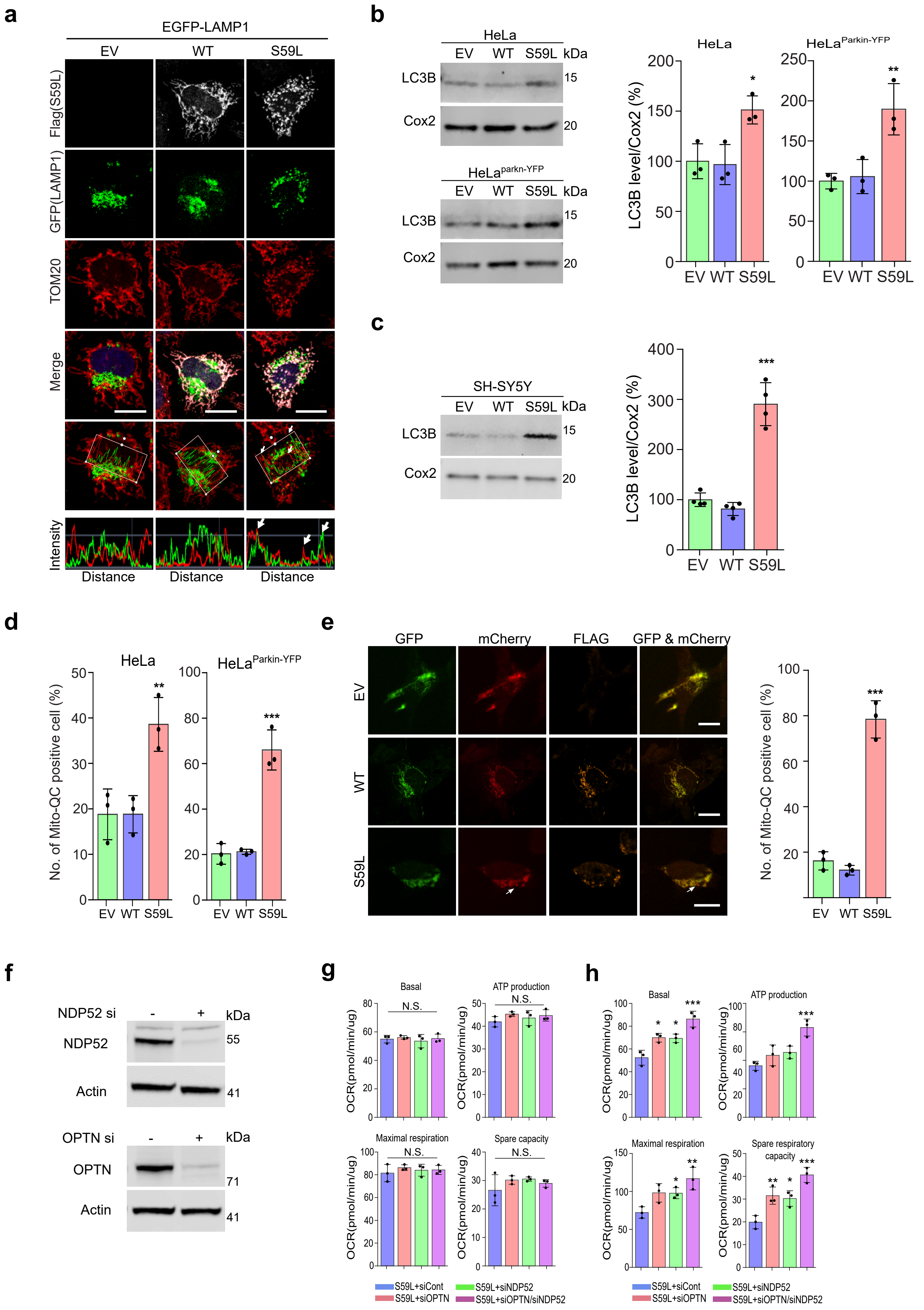
a
EGFP-LAMP1
EV
WT
S59L
Flag(S59L)
GFP(LAMP1)
TOM20
Merge
Intensity
Distance
b
HeLa
EV WT S59L kDa
LC3B
Cox2
15
20
HeLa^parkn-YFP
HeLa^Parkin-YFP
LC3B level/Cox2 (%)
c
SH-SY5Y
d
No. of Mito-QC positive cell (%)
e
GFP
mCherry
FLAG
GFP & mCherry
f
NDP52 si
NDP52
Actin
OPTN si
OPTN
55
41
71
g
Basal
ATP production
Maximal respiration
Spare capacity
N.S.
OCR(pmol/min/ug)
S59L+siCont
S59L+siOPTN
S59L+siNDP52
S59L+siOPTN/siNDP52
h
Spare respiratory capacity

Supplementary Fig. 6 Effect of *CHCHD10^{S59L}* on mitophagy. (**a**) HeLa cells were transfected with *EGFP-LAMP1* and FLAG-tagged *CHCHD10^{S59L}*. After 24 hours of transfection, cells were immunostained with antibodies against FLAG and TOM20. Graphs show the fluorescence intensity profiles of EGFP-LAMP1 and TOM20 along the regions boxed in white. Arrows indicate highly LAMP1-S59L merged regions. Representative images from 2 independent experiments are shown. Scale bar = 20 μm. **(b)** HeLa, HeLa$^{YFP\text{-}parkin,}$ and (**c**) SH-SY5Y cells were transfected with *CHCHD10^{WT}* or *CHCHD10^{S59L}*. After 48 hours, mitochondria were isolated. Immunoblotting was conducted with anti-LC3B and anti-Cox2 (loading controls) antibodies. Data shown are mean ± SD (one-way ANOVA and *posthoc* Dunnett test, two-sided, *p = 0.0197, **p = 0.0054, ***p = 6.9e-06, compared to vector control; n = 3 independent experiments). (**d**) Quantification of Fig. 6i, j. Data shown are mean ± SD (one-way ANOVA and *posthoc* Dunnett test, two-sided, **p = 0.0065, ***p = 0.00013, compared to vector control; n = 3 independent experiments, >30 cells were counted for each group). (**e**) SH-SY5Y cells were co-transfected with *CHCHD10^{WT}* or *CHCHD10^{S59L}* and Mito-QC. Cells with mito-lysosomes (GFP^{-}/mCherry^{+}) were analyzed. Data shown are mean ± SD (one-way ANOVA and *posthoc* Dunnett test, two-sided, ***p = 1.5e-05, n = 3 independent experiments with ≥ 40 cells for each group). Scale bar = 10 μm. (**f**) HeLa cells were transfected with siRNAs targeting *NDP52* or *OPTN* or control siRNA. Immunoblotting confirmed RNAi-mediated knockdown of target genes. Representative images from 2 independent experiments are shown. (**g**) HeLa cells and (**h**) HeLa$^{YFP\text{-}Parkin}$ cells were transfected with siRNAs targeting *NDP52* and/or and *OPTN*. Mitochondrial respiration was measured by Seahorse XF Cell Mito Stress tests at 24 hours after CHCHD10^{S59L} transfection. Graphs of a single representative experiment are shown (mean ± SD). Actual statistical analyses were performed with 3 independent experiments. Data were

compared with one-way ANOVA and *posthoc* Dunnett test, two-sided, *$p = 0.0129$ (siOPTN), 0.0108 (siNDP52), ***$p = 0.00015$ (siOPTN/ siNDP52) in basal level, *$p = 0.0479$, **$p = 0.0028$ in maximal level, ***$p = 0.00078$ in ATP production, **$p = 0.01385$, *$p = 0.00732$ and ***$p = 0.00016$ in spare respiratory capacity. Detailed information on statistical analyses is available in Supplementary Fig. 9j, k.

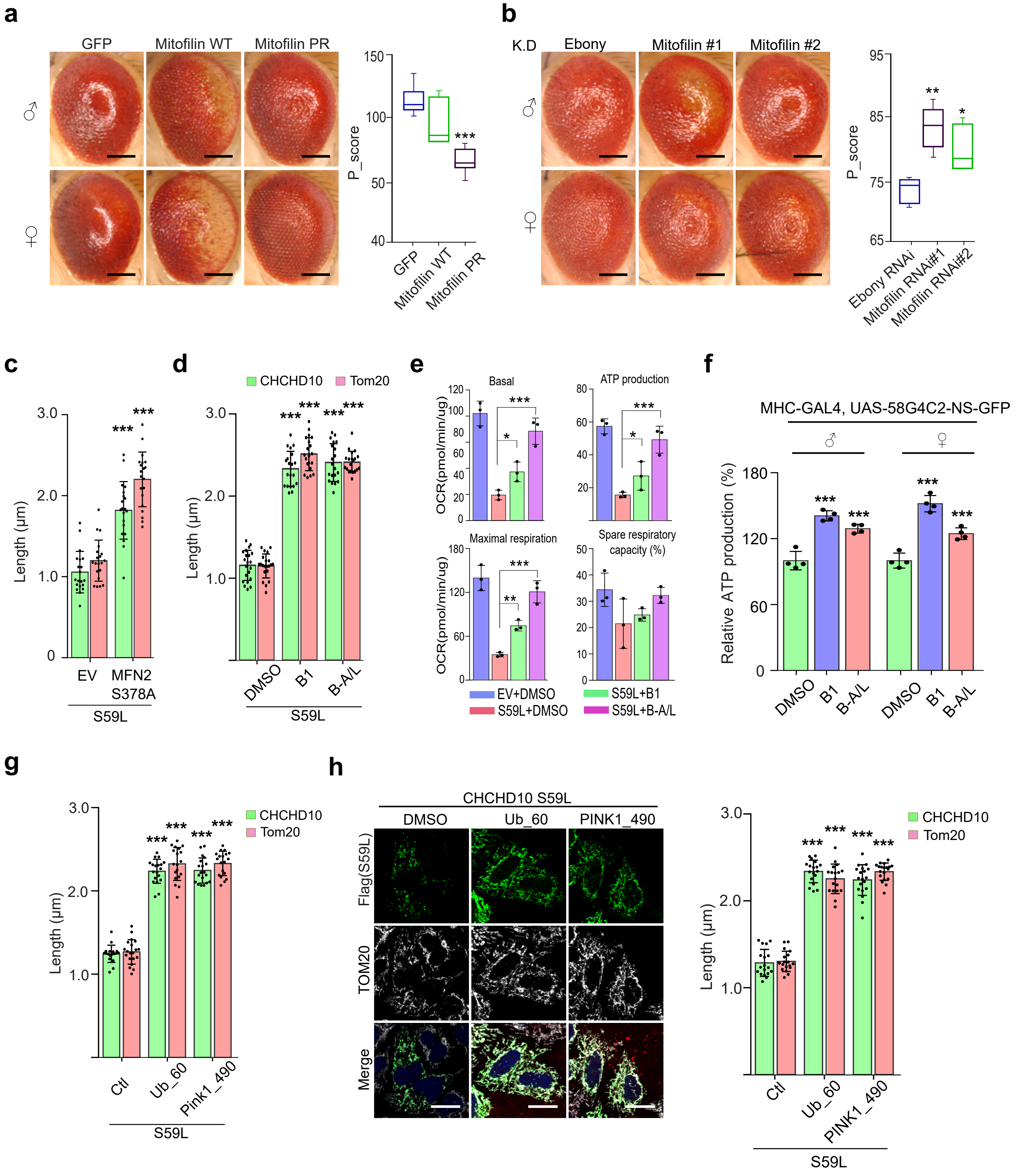

a
GFP
Mitofilin WT
Mitofilin PR
♂
♀
P_score
150
100
50
40
***
GFP
Mitofilin WT
Mitofilin PR
b
K.D
Ebony
Mitofilin #1
Mitofilin #2
95
85
75
65
**
*
Ebony RNAi
Mitofilin RNAi#1
Mitofilin RNAi#2
c
Length (μm)
3.0
2.0
1.0
0
EV
MFN2 S378A
S59L
d
CHCHD10
Tom20
DMSO
B1
B-A/L
e
Basal
ATP production
Maximal respiration
Spare respiratory capacity (%)
OCR(pmol/min/ug)
EV+DMSO
S59L+DMSO
S59L+B1
S59L+B-A/L
f
MHC-GAL4, UAS-58G4C2-NS-GFP
Relative ATP production (%)
180
120
60
g
Ctl
Ub_60
Pink1_490
h
CHCHD10 S59L
DMSO
Ub_60
PINK1_490
Flag(S59L)
TOM20
Merge

Supplementary Fig. 7 Mitigation of *CHCHD10*^{S59L}-induced toxicity by inhibiting PINK1 activity. (**a**) Overexpression of nonphosphorylatable *mitofilin* partially rescued *C2C10H*^{S81L}-induced rough eye phenotypes. Interestingly, overexpression of WT mitofilin cause mixed effects on eye degeneration, slightly better in anterior regions but much worse in posterior regions. Boxes indicate median and 25th and 75th percentiles, and bars indicate the highest and lowest values (one-way ANOVA and *posthoc* Dunnett test, two-sided, ***p = 0.00085; n = 5–6 for each group from 3 independent experiments). Scale bar = 200 μm. (**b**) RNAi-mediated knockdown of *Mitofilin* enhanced the *C2C10H*^{S81L} phenotype. Ebony RNAi, V45688; Mitofilin RNAi #1, V47616; Mitofilin RNAi #2, V47615. Boxes indicate median and 25th and 75th percentiles, and bars indicate the highest and lowest values (one-way ANOVA and *posthoc* Dunnett test, two-sided, **p = 0.0017, *p = 0.0369; n = 5–6 from 3 independent experiments). Scale bar = 200 μm. (**c**) Mitochondrial and CHCHD10 signal length were analyzed from *MFN2*^{S378A} and *CHCHD10*^{S59L} transfected cells. Data shown are mean ± SD (two-sided t-test, ***p = 5.23e-10 for CHCHD10 and 1.9e-13 for Tom20; n = 20 cells from 3 independent experiments). (**d**) The effect of MFN2 agonists on mitochondrial and CHCHD10 signal length in HeLa cells expressing *CHCHD10*^{S59L}. Data shown are mean ± SD (one-way ANOVA and *posthoc* Dunnett test, two-sided, ***p < 2e-16 for both B1 and B-A/l in Tom20, compared to DMSO; n = 20-21 cells from 3 independent experiments). (**e**) HeLa cells were transfected with FLAG-tagged *CHCHD10*^{S59L} and treated with the MFN2 agonists B1 (50 nM) or B-A/L (5 nM) for 24 hours. Mitochondrial respiration was measured with the Seahorse XF Cell Mito Stress test kit. Graphs of a single representative experiment are shown (mean ± SD). Actual statistical analyses were performed with 4 independent experiments (one-way ANOVA and *posthoc* Dunnett test, two-sided, *p = 0.0948, ***p = 0.0004 for basal level, **p = 0.0047, ***p = 7.1e-

05 for maximal level, *p = 0.1457, **p = 0.0019 for ATP production; detailed information on statistical analyses is available in Supplementary Fig. 9i). (**f**) ATP levels in thoraxes from 10-day-old flies expressing *C9orf72* with expanded GGGGCC repeats fed with MFN2 agonists (each 10 μM). DMSO (0.1%) was used as vehicle control. Data shown are mean ± SD (one-way ANOVA and *posthoc* Dunnett test, two-sided, ***p = 9.7e-06, 0.00014 in male, ***p = 2.8e-06, 0.00097 in female for B1 and B-A/L treatment, respectively; n = 4 independent experiments). (**g**) Effects of PINK1 inhibitors on mitochondrial length in HeLa cells expressing $CHCHD10^{S59L}$. Data shown are mean ± SD (one-way ANOVA and *posthoc* Dunnett test, two-sided, *** p < 2e-16 from all groups; n = 20-21 cells from 3 independent experiments). (**h**) HeLa cells were pretransfected for 24 hours with FLAG-tagged $CHCHD10^{S59L}$ and then treated with the peptide inhibitors PINK1_490 (0.15 μg/ml) and Ub_60 (0.15 μg/ml). DMSO was used as vehicle control. Cells were visualized with antibodies against FLAG and TOM20. Data shown are mean ± SD (one-way ANOVA and *posthoc* Dunnett test, two-sided, ***p < 3e-16 for all groups; n = 20 cells from 3 independent experiments). Scale bar = 20 μm.

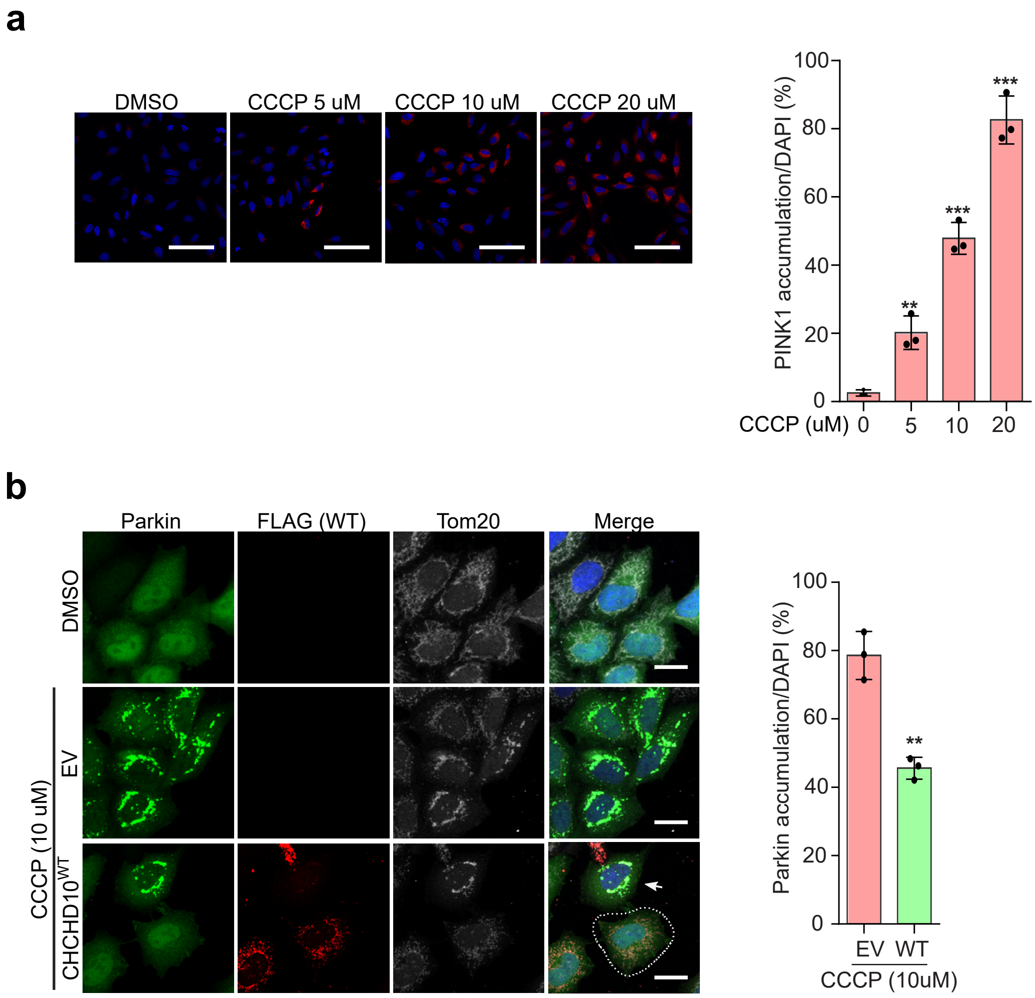
a
DMSO
CCCP 5 uM
CCCP 10 uM
CCCP 20 uM
PINK1 accumulation/DAPI (%)
100
80
60
40
20
0
**
***
***
CCCP (uM) 0 5 10 20
b
Parkin
FLAG (WT)
Tom20
Merge
DMSO
EV
CCCP (10 uM)
CHCHD10WT
Parkin accumulation/DAPI (%)
100
80
60
40
20
0
**
EV WT
CCCP (10uM)

Supplementary Fig. 8 Effect of *CHCHD10* on CCCP-induced PINK1/parkin accumulation. (**a**) To titrate CCCP dosage for PINK1 accumulation, $\text{HeLa}^{\text{PINK1-V5-His}}$ cells treated with multiple CCCP concentrations for 6 hours and then analyzed with a V5 antibody (red) and DAPI (blue). The percentage of PINK1-positive cells was calculated from the number of DAPI^{+} cells. For each sample, ≥300 cells were counted from 3 independent experiments (two-sided *t*-test, $**p$ = 0.0057, $***p$ = 3.1e-05, 2.6e-14 for 5, 10 and 20 μM CCCP treated group, respectively). Scale bar = 100 μm. (**b**) $\text{HeLa}^{\text{YFP-Parkin}}$ cells transfected with EV or FLAG-tagged $CHCHD10^{WT}$ were treated with CCCP (10 μM) for 6 hours. Cells were analyzed with anti-FLAG (red) antibody, YFP (green), and DAPI (blue, nucleus) to visualize CHCHD10 and parkin proteins. Arrow indicates parkin accumulated in a nontransfected cell neighboring a *CHCHD10*-transfected cell (white dashed line) (two-sided *t*-test, $**p = 0.00178$; $n = 3$ independent experiments, with > 200 cells). Scale bar = 20 μm.

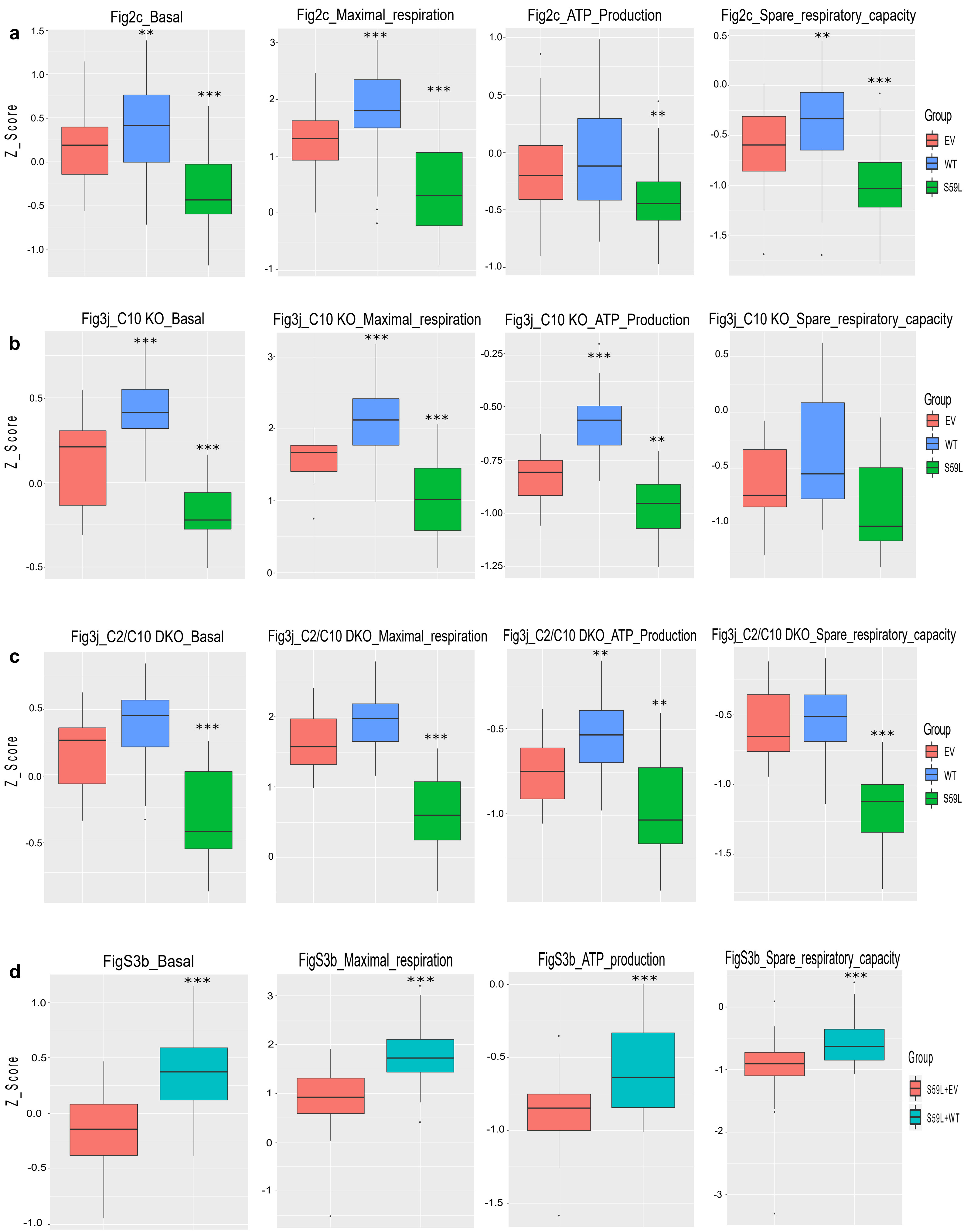
a
Fig2c_Basal
Fig2c_Maximal_respiration
Fig2c_ATP_Production
Fig2c_Spare_respiratory_capacity
b
Fig3j_C10 KO_Basal
Fig3j_C10 KO_Maximal_respiration
Fig3j_C10 KO_ATP_Production
Fig3j_C10 KO_Spare_respiratory_capacity
c
Fig3j_C2/C10 DKO_Basal
Fig3j_C2/C10 DKO_Maximal_respiration
Fig3j_C2/C10 DKO_ATP_Production
Fig3j_C2/C10 DKO_Spare_respiratory_capacity
d
FigS3b_Basal
FigS3b_Maximal_respiration
FigS3b_ATP_production
FigS3b_Spare_respiratory_capacity
Z_Score
Group
EV
WT
S59L
S59L+EV
S59L+WT

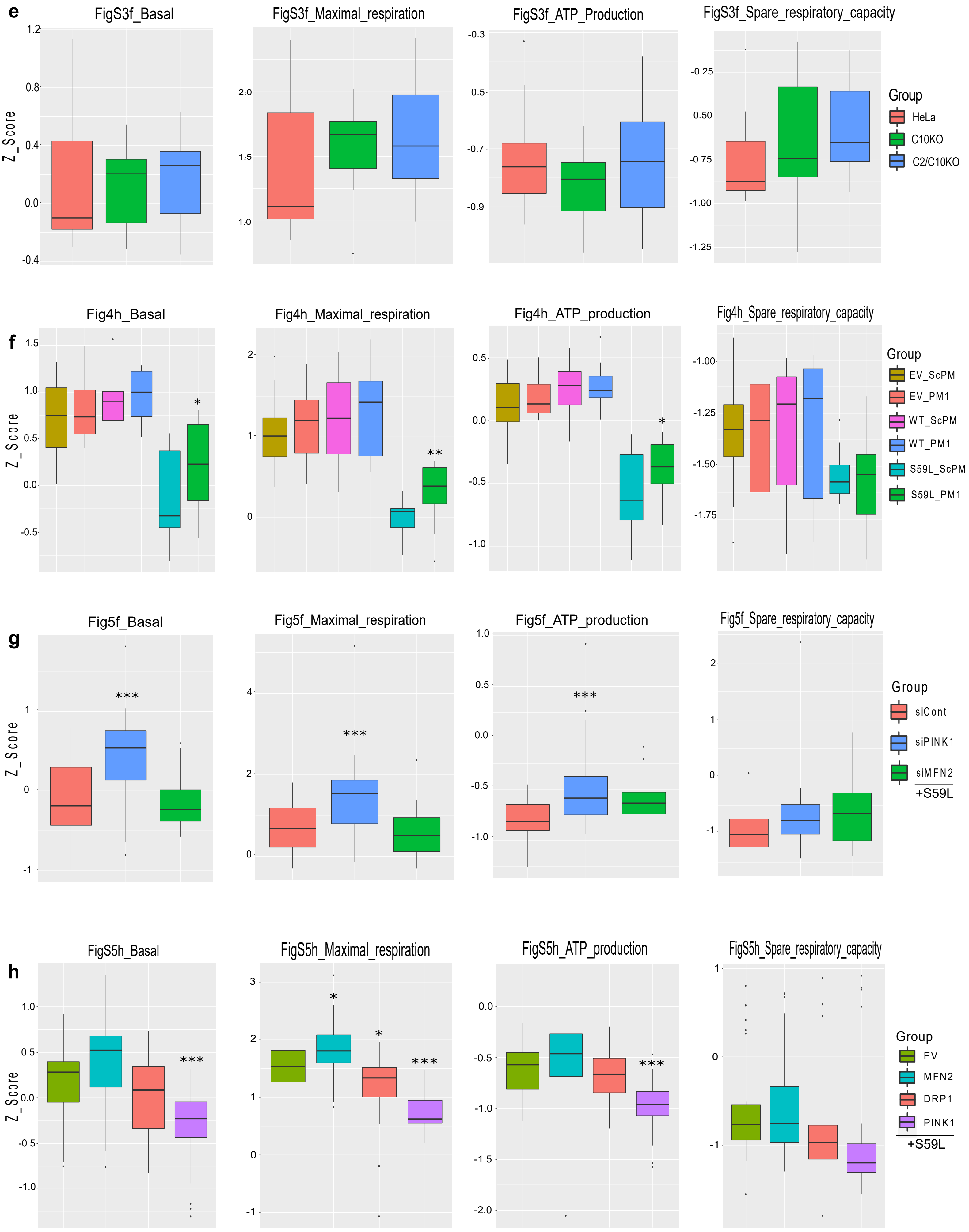

e
FigS3f_Basal
FigS3f_Maximal_respiration
FigS3f_ATP_Production
FigS3f_Spare_respiratory_capacity
Z_Score
Group
HeLa
C10KO
C2/C10KO
f
Fig4h_Basal
Fig4h_Maximal_respiration
Fig4h_ATP_production
Fig4h_Spare_respiratory_capacity
Z_Score
Group
EV_ScPM
EV_PM1
WT_ScPM
WT_PM1
S59L_ScPM
S59L_PM1
g
Fig5f_Basal
Fig5f_Maximal_respiration
Fig5f_ATP_production
Fig5f_Spare_respiratory_capacity
Z_Score
Group
siCont
siPINK1
siMFN2
+S59L
h
FigS5h_Basal
FigS5h_Maximal_respiration
FigS5h_ATP_production
FigS5h_Spare_respiratory_capacity
Z_Score
Group
EV
MFN2
DRP1
PINK1
+S59L

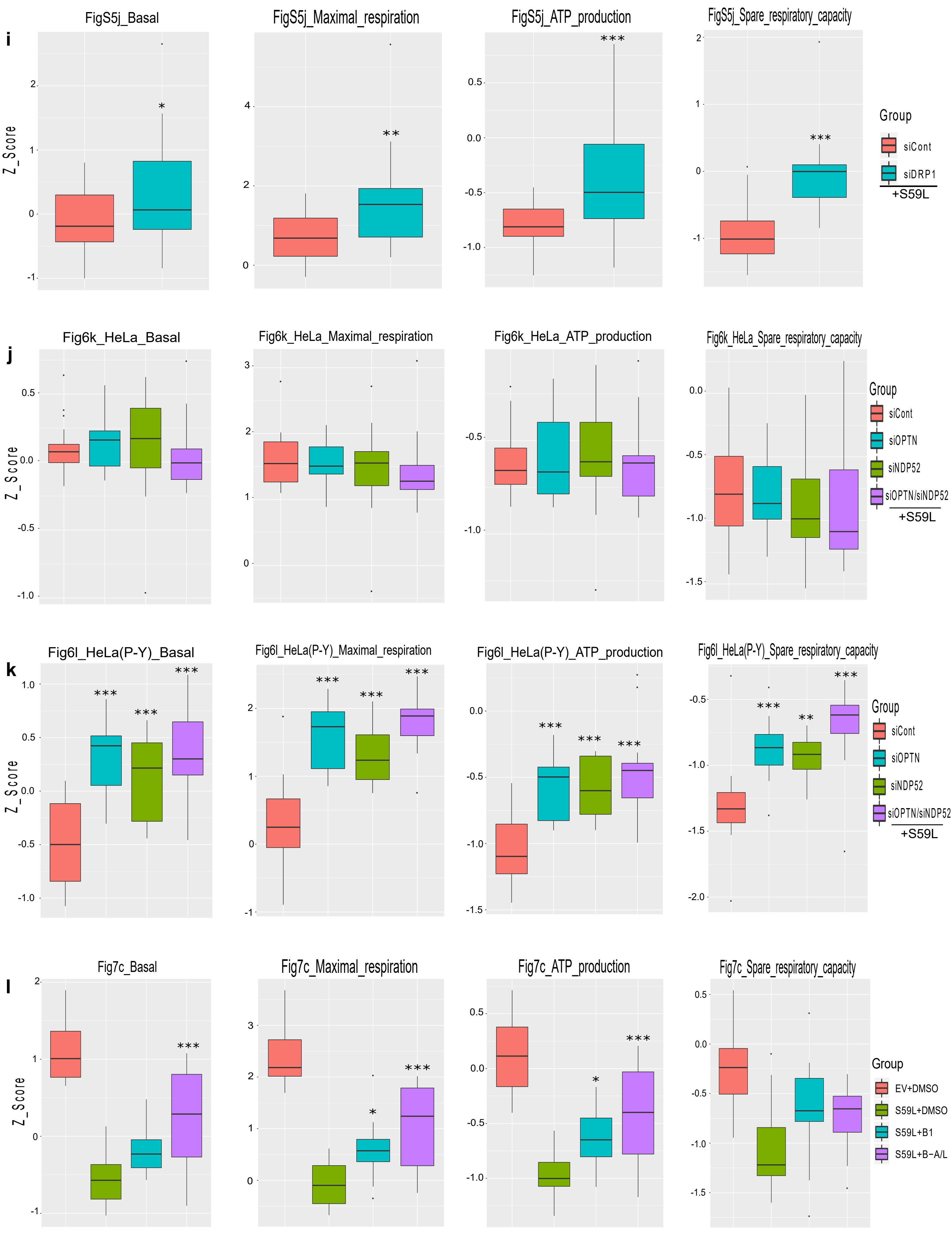
i
FigS5j_Basal
FigS5j_Maximal_respiration
FigS5j_ATP_production
FigS5j_Spare_respiratory_capacity
Z_Score
Group
siCont
siDRP1
+S59L
j
Fig6k_HeLa_Basal
Fig6k_HeLa_Maximal_respiration
Fig6k_HeLa_ATP_production
Fig6k_HeLa_Spare_respiratory_capacity
Group
siCont
siOPTN
siNDP52
siOPTN/siNDP52
+S59L
k
Fig6l_HeLa(P-Y)_Basal
Fig6l_HeLa(P-Y)_Maximal_respiration
Fig6l_HeLa(P-Y)_ATP_production
Fig6l_HeLa(P-Y)_Spare_respiratory_capacity
Group
siCont
siOPTN
siNDP52
siOPTN/siNDP52
+S59L
l
Fig7c_Basal
Fig7c_Maximal_respiration
Fig7c_ATP_production
Fig7c_Spare_respiratory_capacity
Group
EV+DMSO
S59L+DMSO
S59L+B1
S59L+B−A/L

Supplementary Fig. 9 Statistical analyses of S**eahorse assays.** Z-Scores were calculated by using oxygen consumption rate (OCR) values from each plate. Z-Score = (OCR value – mean of OCR values)/standard deviation of OCR values. Boxes indicate medians and 25th and 75th percentiles. Bars indicate the highest and lowest values. (**a–d, f–i, k)** one-way ANOVA and *posthoc* Dunnett tests (two-sided) were used to test statistical significance. (**e, j, l**) Two-sided *t*-tests were used to test statistical significance. Detailed statistical value is listed in Supplementary table 3.

Supplementary Table 1. ***Drosophila*** **lines used in the study.**

**Fly Gene**	**Human Gene**	**Stock No. (RNAi or LOF)**	**Stock No. (UAS or Duplicate)**	**Note**
Pink1	*PINK1*	V21860, V109614, B55886, B51650	*UAS*-dPINK1[45]	RNAi: suppressor, *UAS*: enhancer
Park	*PRKN*	V47637, V104363, B38333	*UAS*-Parkin[45]	RNAi: suppressor, *UAS*: enhancer
Marf	*MARF*	B31157, B55189, V40478, V105261	*UAS*-Marf[45], B32267, B30293	RNAi: enhancer, *UAS*: suppressor
Drp1	*DNM1L*	B27682, B51483, V44155, V44156	B51647	RNAi: enhancer, *UAS*: enhancer (lethal)
ND-42	*NDUFA10*		B58467	*UAS*: No effect
sicily	*NDUFAF6*		B58465, B67141	*UAS*: No effect
Mitofilin	*IMMT*	V47615, V47616, V106757	*UAS*-Mitofilin[51], *UAS*-MitofilinPR[51]	RNAi: enhancer, *UAS*-PR: suppressor
Miro	*RHOT1*	B27695, B43973	*UAS*-dMiro[74], *UAS*-Myc-dMiro[74]	RNAi: enhancer, *UAS*: enhancer
ari-1	*ARIH1*	B29416, B1538	B30756	RNAi: No effect, Duplicate: No effect
mul1	*MUL1*	V109808		RNAi: No effect
Dmel\CG9855	*MARCH5*	V105711		RNAi: No effect
TER94	*VCP*	V24354	*UAS*-Ter94[45]	Both: enhancer
Atg1	*ULK1*	B26731, B35177, B44034	B51654, B51655	RNAi: enhancer, *UAS*: enhancer (lethal)
ref(2)P	*SQSTM1*	B33978, B36111		RNAi: No effect
mib1	*MIB1*	B27320		RNAi: No effect
mib2	*MIB1*	B57833		RNAi: No effect
CG5059	*BNIP3*	B42494		RNAi: No effect
key	*IKBKG*	B35572, B57759		RNAi: No effect
mud	*NUMA1*	B28074, B35044, B38190		RNAi: No effect
tango11	*MFF*	B36281		Deletion mutant: No effect
TBPH	*TARDBP*		*UAS*-hTDP-43 M337V[40]	*UAS*-Disease-causing mutant: lethal
TBPH	*TARDBP*		Gene replacement: WT, G294A, and M337V	G294A and M337V: enhancer

Supplementary Table 2. Primer sequences for quantitative RT-PCR.

Primer Name	Primer Sequence
**PINK1**	Forward : 5'-TAC CAG TGC ACC AGG AGA AG-3'<br>Reverse : 5'-GCT TGG GAC CTC TCT TGG AT-3'
**B2M**	Forward: 5'-GTG GCC CTT AGC TGT GCT CG-3'<br>Reverse: 5'-ACC TGA ATG CTG GAT AGC CTC-3'
**28S**	Forward: 5'-ACC TGT CTC ACG ACG GTC TA-3'<br>Reverse: 5'-GCG TTG GAT TGT TCA CCC AC-3'

Supplementary Table 3. Summary of Seahorse assays.

Figures	Total number of assay sets	Parameters	*p* value (*t*-test)	*p* value (ANOVA)	*p* value (posthoc analysis)
Fig. S9a (Fig. 2c)	11	Basal	—	2.29E-16	[S59L - EV: 1.2e-08], [WT - EV: 0.0022]
		Maximal respiration	—	<2e-16	[S59L - EV: 5.7e-09], [WT - EV: 0.00059]
		ATP production	—	8.79E-07	[S59L - EV: 0.0013], [WT - EV: 0.0805]
		Spare respiratory capacity	—	4.08E-11	[S59L - EV: 1.6e-05], [WT - EV: 0.0095]
Fig. S9b, Fig. S9c (Fig. 3j)	CHCHD10 KO (**C10 KO**): 3, C2&C10 KO (**C2/C10 KO**): 5	Basal	—	**C10 KO**: 4.87e-12, C**2/C10 KO**: 2.48e-09	[C**10 KO**: S59L - EV: 2.6e-05, WT - EV: 1.0e-04]; [C**2/C10 KO**: S59L - EV: 8.8e06, WT - EV: 0.0623]
		Maximal respiration	—	**C10 KO**: 1.45e-09, C**2/C10 KO**: 4.94e-13	[C**10 KO**: S59L - EV: 0.00056, WT - EV: 0.00067]; [C**2/C10 KO**: S59L - EV: 1.4e-09, WT - EV: 0.1644]
		ATP production	—	**C10 KO**: 3.11e-12, C**2/C10 KO**: 7.05e-08	[C**10 KO**: S59L - EV: 0.0041, WT - EV: 3.7e-07]; [C**2/C10 KO**: S59L - EV: 0.0012, WT - EV: 0.0094]
		Spare respiratory capacity	—	**C10 KO**: 0.00209, C**2/C10 KO**: 3.94e-12	[C**10 KO**: S59L - EV: 0.1341, WT - EV: 0.1065]; [C**2/C10 KO**: S59L - EV: 4.6e-10, WT - EV: 0.8313]
Fig. S9d (Fig. S3c)	8	Basal	1.11E-11	—	—
		Maximal respiration	3.78E-11	—	—
		ATP production	9.50E-07	—	—
		Spare respiratory capacity	8.26E-06	—	—
Fig. S9e (Fig. S3h)	3	Basal	—	0.966	C10KO - HeLa: 1.0000, C2/C10KO - HeLa: 0.9648
		Maximal respiration	—	0.194	C10KO - HeLa: 0.3930, C2/C10KO - HeLa: 0.1256
		ATP production	—	0.109	C10KO - HeLa: 0.1526, C2/C10KO - HeLa: 0.9975
		Spare respiratory capacity	—	0.136	C10KO - HeLa: 0.3281, C2/C10KO - HeLa: 0.0838
Fig. S9f (Fig. 4h)	4	Basal	0.0376	—	—
		Maximal respiration	0.0069	—	—
		ATP production	0.0413	—	—

Figures	Total number of assay sets	Parameters	p value (t-test)	p value (ANOVA)	p value (posthoc analysis)
		Spare respiratory capacity	0.6862	—	—
Fig. S9g (Fig. 5f)	SiCont + S59L: 8, siPINK1 + S59L: 8, siMFN2 + S59L: 3	Basal	—	3.53E-05	S59L+siMFN2' - 'S59L+siCont': 0.86400; 'S59L+siPINK1' - 'S59L+siCont': 0.00012
		Maximal respiration	—	0.000353	S59L+siMFN2' - 'S59L+siCont': 0.8775; 'S59L+siPINK1' - 'S59L+siCont': 0.00093
		ATP production	—	8.16E-05	S59L+siMFN2' - 'S59L+siCont': 0.0787; 'S59L+siPINK1' - 'S59L+siCont': 3.1e-05
		Spare respiratory capacity	—	0.106	S59L+siMFN2' - 'S59L+siCont': 0.1723; 'S59L+siPINK1' - 'S59L+siCont': 0.1134
Fig. S9h (Fig. S5h)	6	Basal	—	3.19E-07	DRP1+S59L' - 'EV+S59L': 0.58731, 'MFN2+S59L' - 'EV+S59L': 0.09216, 'PINK1+S59L' - 'EV+S59L': 0.00039
		Maximal respiration	—	5.32E-13	DRP1+S59L' - 'EV+S59L': 0.0239, 'MFN2+S59L' - 'EV+S59L': 0.0419, 'PINK1+S59L' - 'EV+S59L': 1.1e-07
		ATP production	—	6.37E-07	DRP1+S59L' - 'EV+S59L': 0.79002, 'MFN2+S59L' - 'EV+S59L': 0.28146, 'PINK1+S59L' - 'EV+S59L': 0.00012
		Spare respiratory capacity	—	0.0725	DRP1+S59L' - 'EV+S59L': 0.4668, 'MFN2+S59L' - 'EV+S59L': 0.9687, 'PINK1+S59L' - 'EV+S59L': 0.1194
Fig. S9i (Fig. S5j)	siCont + S59L: 8, siDRP1 + S59L: 3	Basal	0.04	—	—
		Maximal respiration	0.00122	—	—
		ATP production	0.00016	—	—
		Spare respiratory capacity	1.81E-06	—	—
Fig. S9j, Fig. S9k (Fig. 6k,l)	**HeLa**: 3, **HeLa$^{YFP\text{-}Parkin}$**: 3	Basal	—	**HeLa**: 0.801, **HeLa(P-Y)**: 6.67e-07	[**HeLa**: 'siNDP52+S59L' - 'siCont+S59L': 0.9960, 'siOPTN+S59L' - 'siCont+S59L': 0.9966, 'siOPTN/siNDP52+S59L' - 'siCont+S59L': 0.8262], [**HeLa(P-Y)**: 'siNDP52+S59L' - 'siCont+S59L': 0.00036, 'siOPTN+S59L' - 'siCont+S59L': 5.6e-06, 'siOPTN/siNDP52+S59L' - 'siCont+S59L': 1.6e-06]

Figures	Total number of assay sets	Parameters	*p* value (*t*-test)	*p* value (ANOVA)	*p* value (posthoc analysis)
		Maximal respiration	—	**HeLa**: 0.763, **HeLa(P-Y)**: 3.3e-09	[**HeLa**: 'siNDP52+S59L' - 'siCont+S59L': 0.8388, 'siOPTN+S59L' - 'siCont+S59L': 0.9798, 'siOPTN/siNDP52+S59L' - 'siCont+S59L': 0.6220], [**HeLa(P-Y)**: 'siNDP52+S59L' - 'siCont+S59L': 2.9e-05, 'siOPTN+S59L' - 'siCont+S59L': 2.8e-07, 'siOPTN/siNDP52+S59L' - 'siCont+S59L': 3.2e-09]
		ATP production	—	**HeLa**: 0.948, **HeLa(P-Y)**: 1.59e-06	[**HeLa**: 'siNDP52+S59L' - 'siCont+S59L': 0.9813, 'siOPTN+S59L' - 'siCont+S59L': 0.9805, 'siOPTN/siNDP52+S59L' - 'siCont+S59L': 0.9944], [**HeLa(P-Y)**: 'siNDP52+S59L' - 'siCont+S59L': 0.00018, 'siOPTN+S59L' - 'siCont+S59L': 5.5e-05, 'siOPTN/siNDP52+S59L' - 'siCont+S59L': 1.8e-06]
		Spare respiratory capacity	—	**HeLa**: 0.7, **HeLa(P-Y)**: 5.22e-06	[**HeLa**: 'siNDP52+S59L' - 'siCont+S59L': 0.5706, 'siOPTN+S59L' - 'siCont+S59L': 0.8872, 'siOPTN/siNDP52+S59L' - 'siCont+S59L': 0.6703], [**HeLa(P-Y)**: 'siNDP52+S59L' - 'siCont+S59L': 0.00441, 'siOPTN+S59L' - 'siCont+S59L': 0.00064, 'siOPTN/siNDP52+S59L' - 'siCont+S59L': 2.1e-06]
Fig. S9l (Fig. 7c)	4	Basal	—	1.43E-03	B1+S59L' - 'S59L+DMSO': 0.12592, 'B-A/L+S59L' - 'S59L+DMSO': 0.00064
		Maximal respiration	—	1.49E-03	'B1+S59L' - 'S59L+DMSO': 0.02872, 'B-A/L+S59L' - 'S59L+DMSO': 0.00076
		ATP production	—	1.09E-03	'B1+S59L' - 'S59L+DMSO': 0.0312, 'B-A/L+S59L' - 'S59L+DMSO': 0.00052
		Spare respiratory Capacity	—	0.107	'B1+S59L' - 'S59L+DMSO': 0.0882 , 'B-A/L+S59L' - 'S59L+DMSO': 0.1679
